# The perceptions of newly qualified nurses on the guidance by preceptors towards becoming experts in nursing

**DOI:** 10.4102/curationis.v44i1.2205

**Published:** 2021-11-24

**Authors:** Warriodene Hansen

**Affiliations:** 1 Department of Nursing, Faculty of Health Sciences, University of South Africa, Cape Town, South Africa

**Keywords:** expert, hospital, novice, nurse, preceptor, preceptorship, registered nurse

## Abstract

**Background:**

The new role as professional nurse can be a difficult transition for the new qualified nurses. During this time, factors such as not being well prepared, working without supervision and a lack of guidance can be the result of a difficult transition.

**Objectives:**

The purpose of this study is to assess the perceptions of newly qualified nurses on the guidance given by their preceptors towards becoming experts in practice at a Level II regional hospital in the Western Cape.

**Method:**

A non-experimental quantitative descriptive design was followed. Collection of data was done by means of a questionnaire, designed by the researcher, using a cross-sectional research method. Non-probability sampling produced a sample of 162 nurses comprising registered nurses (48.2%), enrolled nurses (32.7%), and enrolled nursing auxiliaries (19.1%). Statistical analysis was performed using the Statistical Analysis System (SAS), version 9.3.

**Results:**

The results of the research study indicated that respondents had more positive experiences than negative ones. The respondents indicated that for the role and characteristics of the preceptor, expectations were met for knowledgeability, professionalism and contribution to team work. Furthermore, the results indicated that the respondents would recommend preceptorship.

**Conclusion:**

Preceptorship is one of the major interventions available to support newly qualified nurses by easing the transition from student to practicing nurse and reducing the theory-practice gap. The findings emphasised the importance of ongoing support programmes for nurses after obtaining a new qualification or/and being a new nurse.

## Introduction

The need to support employed staff who recently graduated led to the development of preceptorship. In the nursing profession, the past and rise of preceptorship can be tracked to Florence Nightingale, who wrote in 1882 that first year nurses’ ‘practical and technical education’ must be supported by nurses who are skilled to train (Lennox, Skinner & Foureur 2008:9). According to Jones (2015:23), the preceptor model evolved as a result of matters recognised with the transition of newly registered nurses from educationally prepared nursing programmes. In the United Kingdom (UK), the form of support for newly qualified nurses has been accepted as preceptorship since 1990 (Whitehead et al. 2013:370). Since 2000, numerous models for nurse residency programmes and other transition programmes have been developed and almost every report included precepted clinical experience in the models (Blegen et al. 2015:642). Clinical practice is thus agreed to be an essential part of all healthcare professions in which preceptors play a vital role (Bengtsson & Carlson 2015:2).

Globally, nursing forms the biggest cluster of the healthcare profession (Chong et al. 2014:191) and nurses are is responsible for up to 80% of the care of patients (Sodeify, Vanaki & Mohammadi 2013:191). In addition, Rogan (2014:13) is of the opinion that the basic nursing education is insufficient to prepare the novice nurses to assume and fulfil the role of an expert. Therefore, globally, healthcare professionals have recognised that guidance of novice nurses to adapt to the challenging and continuously changing healthcare environment is a vitally important component of providing high quality and evidence-based care.

The concept of preceptorship is similar in countries such as New Zealand, Australia, United States of America (US) and the UK (Lennox et al. 2008:9). According to Jones (2015:23), preceptorship can take place in a number of situations, such as providing support to undergraduate nursing students and new entrants to practice, orientating registered nurses to the workplace or as part of a ‘Competency Assessment Programme’ process. Whitehead et al. (2013:377), indicated that properly resourced and organised preceptorship is a positive and vital experience for newly qualified nurses and their employers. Padayachee (2014:51) suggested that nursing managers support and agree that preceptorship is vitally important to the practical ability of nursing undergraduates. Whilst Rikhotso, Williams and De Wet (2014:5) concluded that students relish learning and working under the supervision of an expert registered nurse for their effective professional growth. According to Whitehead et al. (2013:376), methods prescribed to improve newly qualified nurses’ confidence include ensuring that a preceptor supports them and that they have a programme of preceptorship.

### Expectations and perceptions of preceptorship programmes

Once enrolled in any programme, a person has some expectations and perceptions of the specific programme. The following literature discusses and illustrates examples of expectations and perceptions of preceptorship programmes. There are many programmes aimed at the development of personnel, as there are for preceptorship, and each one is chosen to address a need. A review of literature findings has recommended that preceptorship programmes lead to improved satisfaction of newly graduated nurses and also staff retention. These programmes may help overcome the worldwide shortage of nurses and in view of this, preceptors and leaders need to offer a conducive environment in which newly graduated nurses feel content with their workloads and their tasks (Arbabi, Johnson & Forgave 2018:48). Preceptors need to create an environment in which preceptees will excel during their preceptorship programme. That is why the teaching style, knowledge and experience of the preceptor are important to the preceptorship programme. A qualitative study by Dias et al. (2015:53) conducted in Pakistan, interviewing nine students and six preceptors in focus group discussions found that students reported that preceptors are an invaluable resource. Miller, Vivona and Roth (2016:2020) found in their study that a majority of respondents reported positive experiences with their preceptors. Furthermore, in a study in the US with 34 preceptors and 30 novice nurses as participants, Hickerson, Terhaar and Taylor (2016:61) found that the preceptor support programme was associated with positive assessments by the novice nurses of their preceptors’ teaching and support. It can be seen that students appreciate the preceptorship model as an innovative way of teaching pedagogy (Dias et al. 2015:53). In a study conducted in Ethiopia with 109 respondents, Teferra and Mengistu (2017:86) found that of the respondents who are familiar with the clinical preceptorship concept, 96.3% of the respondents were classified as having a favourable attitude towards clinical preceptorship.

## Background

According to Jeggels, Traut and Africa (2013:3) preceptorship was developed in 2009 for a preceptorship training programme for registered professional nurses and, after a review of a pilot programme, the first training of nurses in the Western Cape was offered in July 2010 at the University of the Western Cape, South Africa. This continuous development programme was an 80-h, eight-credit short course that included a clinical component (Jeggels et al. 2013:3). While, Botma, Jeggels and Uys (2012:6) found that a preceptorship programme is now also offered at the University of the Free State (UFS) and with a copious amount of homework. The preceptorship programme is based on the preceptorship module that involves all stakeholders, for example, preceptor, student, university and clinical institution (Botma et al. 2012:9). The authors further added that the UFS, so far, has only trained their staff and was planning to roll out the preceptor training programme further.

Cloete and Jeggels (2014:2) posited that nurse preceptors have an essential role in clinical education and learning. These statements are all supported by Peixoto, Tavares and De Queiros (2014:2039), who added that the role of the preceptor is with new graduates in the hospital or place of work, whilst the lecturer’s role is institutional in the college or educational context. A model that relies on a matured preceptor–nurse relationship using trained preceptors had a positive return on investment and provided additional evidence to support the business case for implementing a transition-to-practice programme to decrease new graduate registered nurse turnover (Silvestre et al. 2017:118). Missen, McKenna and Beauchamp (2014:2431) found that results of their study provided evidence that graduate transition programmes are essential. However, a test for preceptors may be the workload related to being a preceptor, which includes a full nursing assignment in addition to the education of the student/preceptee (Chapman 2017:109).

A preceptor is described as a ‘specialised tutor who gives practical training to the student in the practice setting’ (Cloete & Jeggels 2014:1). In addition, Koetting, Ecuyer and Benz (2015:125) added that a preceptor promotes professional and personal development, along with assisting the transition to advanced practice. According to Ryan and McAllister (2016:268), nurse preceptors are defined as ‘buddies, mentors, facilitators of clinical learning, promoters of professional development and providers of students’ pathways for orientation and socialisation into the nursing discipline’. Owens (2013:2) posited that the term ‘preceptor’ means to tutor, guide and evaluate.

### Role of the nurse preceptor

A large body of nursing education research has repeatedly described the role of nurse preceptor, in particular, as a ‘vital part of nursing students’ clinical learning’ (Ryan & McAllister 2016:267).

The preceptor role includes the following positive factors:

- The role of educator is instituted (Peixoto et al. 2014:2039).
- A preceptor demonstrates technical skills, planning, organisational abilities, priority settings, decision-making, communication skills (Owens 2013:2) and a positive role model (Foote et al. 2014:262).
- Preceptors help with the development of knowledge of clinical skills and professional attributes in nursing, encourage the improvement of critical thinking and problem-solving skills and play a vital role in the transition of nursing students from undergraduate nursing to clinical practice (Cloete & Jeggels 2014:7).
- Preceptors are expected to offer their preceptees the best conditions possible to let them grow in their career (Bengtsson & Carlson 2015:2).
- An important element is the relationship the preceptor develops with the new graduate nurse (Lalonde & Hall 2017:25).
- Preceptors help to build confidence in newly qualified nurses and further develop their knowledge and skills (Lewis & McGowan 2015:41).
- Botma (2016:5) found that preceptors are able to facilitate learning by using a variety of coaching, mentoring and facilitation techniques to identify learning needs, apply assessment skills and have the ability to promote the implementation of best practice guidelines.

### Characteristics of the preceptor

A preceptor must portray certain qualities (Dornblaser et al. 2016:3). The following are some key characteristics, according to various authors:

- The preceptor is perceived as a role model (Bolt et al. 2016:144; Botma 2016:6), teacher and evaluator (Owens 2013:2).
- The role of the preceptor include: the ability to coach, guide, inspire, teach and act as a role model with intrinsic rewards and extrinsic demands (Bengtsson & Carlson 2015:2).
- Nurse preceptors are able to provide assessment on the newly qualified nurses’ development (Ryan & McAllister 2017:268).
- The preceptor possesses the drive to protect, teach and socialise (Hall 2016:26).
- The preceptor serves as an instructor (Bolt et al. 2016:144), as teaching in clinical environments is broadly recognised as a core component of nursing education (Dahlke et al. 2016:145).
- Lalonde and Hall (2017:24) mentioned that it is vital that preceptors display emotional intelligence.

### Conceptual framework

The following literature discusses how Benner (1982:402) used the Dreyfus Model of Skill Acquisition to differentiate between a more skilled and knowledgeable nurse and a less experienced. The five levels of development that she derived from the Dreyfus model are outlined here.

According to Dreyfus and Dreyfus (1980:7) the original model proposes that a student passes through five distinct stages, namely novice, competence, proficiency, expertise and mastery. Each step builds on the previous one as abstract principle is distinguished by experience and the learner gains clinical experience. Benner (1982:402) clarified the five levels of development as outlined here. The steps as they build on each other are described as follows:

#### Level 1: The novice

According to Benner (1982:403) a beginner has little knowledge of the prevailing working conditions. A beginner is trained to work in the given conditions in terms of objective traits. Davis and Maisano (2016:13) stated that the initial novice stage in the model is one in which the individual has had no previous experience with the situation at hand. This stage familiarises a nursing student or an experienced nurse with a new setting (Zerwekh & Garneau 2018:12).

#### Level 2: Advanced beginner

According to Benner (1982:403) an advanced beginner is someone who can do slightly satisfactory work and has handled plenty of actual situations to note the repeated meaningful situational components called facets. This stage starts with the last semester of a nursing student or a graduate nurse (Zerwekh & Garneau 2018:12).

#### Level 3: Competent

According to Benner (1982:404) competence is demonstrated by the nurse starting to see his or her actions in terms of long-term goals. However, competent nurses lack speed and flexibility compared with a person in the proficient phase, the competence phase is experienced with a sense of grasp and skill of nursing practice. As clarified by Davis and Maisano (2016:13), the competent individual is able to work in an efficient and organised manner because of conscious, deliberate planning. This stage starts when an individual has 2 or 3 years’ experience (Zerwekh & Garneau 2018:12).

#### Level 4: Proficient phase

According to Benner (1982:405) the proficient nurse sees situations as a unit, as compared with facets. Furthermore, the author posited that with the experience-based capability, a nurse is able to identify when the anticipated normal is absent and this is because of the experiences of true behaviour. According to Zerwekh and Garneau (2018:12), this stage might apply to nurse clinicians and nursing faculty.

#### Level 5: Expert

According to Benner (1982:405) expert nurses have a profound understanding of circumstances and at this phase, they do not depend on analytical principles to connect their understanding of the situation to a suitable action. The expert has an instinctive grasp of all situations and is able to understand the minor particulars of a problem and provide required care to the specific patient (Hilaire & Sheldon 2015:32). Benner (1982:406) posited that there is much to learn from expert nurses but to define and document skilled nurse performance, a new plan for recognising and describing nursing competencies is needed. This stage is recognised at advanced practice nurse level (Zerwekh & Garneau 2018:12).

From the background provided, it is evident that all the cited authors agree that preceptorship is essential for leading novices on their journey towards becoming experts in their specific field of practice.

### Problem statement

Newly qualified nurses often experience, amongst others, unhappiness at work, stress and anxiety, new job-hunting, absenteeism, negativity, medico-legal issues and trying to pursue a different career. These are experienced often because of the lack of supervision and support, which can be provided by a preceptor. This statement has been supported by various researchers such as Cloete and Jeggels (2014:2), Webster (2016:2) and Zaayman (2016:9), as well as by the comments, discussions and feedback made by colleagues in the clinical field of the hospitals and clinics. Additionally, the need for newly qualified nurses to be clinically and academically supervised and guided is crucial to guarantee that they can subsequently provide safe and competent care, which enables them to continuously grow in the profession from being novices to becoming experts. Given this background and the limited number of preceptors employed in South African hospitals, it can be argued that sufficient preceptor support by academically and clinically prepared nurses is still lacking and that the role of the preceptor is at present vague and needs clarification. This statement is consistent with the views of a number of researchers such as Cloete and Jeggels (2014:6), Hofler and Thomas (2016:135) and Morgan (2017:23). Also, Quek and Shorey (2018:420) assessed the perceptions of preceptorships of newly qualified nurses and found that in the Western Cape, South Africa, the perceived benefits and rewards for the preceptor role and a heavy workload had an impact on commitment to fulfil the preceptor. Despite all the benefits of preceptorship no evidence was found that a preceptorship model was developed in the Western Cape (Makie 2021).

### Purpose of the study

The purpose of this study was to assess the perceptions of newly qualified nurses on the guidance given by their preceptors towards becoming experts in practice at a Level II regional hospital in the Western Cape.

### Research objectives

The objectives of this research were to:

- Assess the support given to newly qualified nurses by preceptors during their transition from novice to expert in a selected Level II regional hospital in the Western Cape.
- Describe the role of the preceptor as viewed by newly qualified nurses in the transition from novice to expert in a selected Level II regional hospital in the Western Cape.

### Significance of the study

The findings of this study could emphasise the importance of ongoing support programmes for all categories of nurses after obtaining a new qualification and being a new nurse. The findings could be of significant assistance to healthcare managers, healthcare workers and others involved in striving to deliver a highly efficient service, a good experience and quality to clients visiting their facilities.

## Research design and method

### Design

A positivist paradigm or approach, using a non-experimental, quantitative, descriptive and cross-sectional design was adopted in this study. The study was descriptive, as it attempted to assess and describe ‘what’ the perceptions of newly qualified nurses are of guidance by preceptors towards becoming experts in practice. There was no manipulation of any variables or interventions, hence, the study was non-experimental. It was cross-sectional, because the data were collected only once. The design was quantitative, because the description was in the form of measurement (numerical).

### Setting

In this study the target population was all the categories of nurses who are registered with the South African Nursing Council and who are employed on a full-time basis by a selected level II regional hospital in the Western Cape. In this study, the population comprised all nurses employed at the time by the selected health care facility. This study recruited newly qualified nurses to participate, which are defined as persons who qualified and were found capable to practice nursing in the way prescribed (South African Nursing Council (SANC) 2005:25). At the time the study was carried out the total number of nursing personnel was 315 as indicated on the employment register of the institution.

### Population and sampling

In this research, convenience sampling was selected by the researcher to select the hospital because of the most readily available persons as participants (Polit & Beck 2017:724).

The study sample consisted of a subsection of the population. Non-probability sampling was used. Purposive sampling was used to choose the nursing population. Based on the criteria that all respondents must be permanently employed at the selected research site and registered at South African Nursing Council (SANC) and completed their training between January 2013 and April 2018. three categories of newly qualified nurses were used. These categories included: registered nurse, enrolled nurse and enrolled auxiliary nurse. This method was chosen because participants were selected based on the purpose of the study.

### Instrument and data collection

According to Creswell and Creswell (2018:147) quantitative approaches focus on carefully measuring variables. A self-administered questionnaire was used to collect data. The instrument was divided into different sections to address questions related to the study problem, aim and research objectives. The advantages of a questionnaire are as follows: large amounts of data can be collected, data for a big sample of participants can be collected by post and is relatively cheap, and questionnaires are easy to compile and analyse. The researcher developed the instrument information obtained from the literature and personal experiences of the researcher.

### Pretesting of the instrument

Pretesting of a instrument allows the research team to identify problems in the design of questions, sequence of questions and procedure for recording responses (Gray et al. 2017:405). The researcher conducted a pre-test study with four registered nurses, who qualified between January 2013 and April 2018, from another hospital. The selected hospital was purposefully chosen for the pre-test. The data gathered throughout the pre-test were analysed cautiously to determine whether the respondents had answered the research questions and to determine if the questions were clear to them. After evaluation of the questionnaire, their suggestions were discussed in a meeting. Adjustments, unclear words and questions were reconsidered and changed.

### Data analysis

According to Polit and Beck (2017:725), the systematic organisation and synthesis of research data analysis is the structured planning and the combination of study data and in quantitative studies, the testing of hypotheses using those data. In this study a research assistant entered the data on a spreadsheet that was then posted to the qualified statistician, who used the Statistical Analysis System (SAS) Software, version 9.3 for data analysis. The level of significance was set at 0.05 and the two-sided test was used for prevalence and the one-sided test of significance for adherence. Data analysis consisted of descriptive analysis and the findings were summarised by means of descriptive analysis. According to Creswell and Creswell (2018:157), this analysis should indicate the means, standard deviations or range of scores.

### Respect for human dignity and autonomy

In this study, respondents had to complete a questionnaire anonymously and could do so at their convenience. Permission was requested from the chief executive officer of the hospital, which was duly granted. Thus, the hierarchy of the system was upheld for gaining permission for the study. The relevant information included details on the purpose, procedures, methods, benefits of the study, and the provision of individual consent. The researcher endorsed the participants’ rights to participate and revoke their decision at any time during this study without providing justification, explanation or incurring any penalty/coercion. The researcher’s email address and telephone number were also included. Thus, through veracity, a trustworthy and transparent relationship was established amongst all the stakeholders. Therefore, the researcher respected the participants’ autonomy by ensuring that they received all the information required to make a knowledgeable decision (Pera & Van Tonder 2017:54).

### Beneficence and non-maleficence

This study aimed to investigate, discover and determine the views of newly qualified nurses on the support by the guidance of a preceptor during their transition from novice to expert; the role of the preceptor; the preceptorship programme; and provide recommendations. In this process, sufficient care was taken in upholding privacy, confidentiality and anonymity of the respondents and the research site. In addition, provision was made for all the stakeholders to avoid inconvenience because of loss of time and economy. Moreover, non-interference during the research in the routine of the nursing and hospital service made this research very practical and feasible. The study fulfilled another objective of non-maleficence by spurning physical, emotional, social, economic and psychosocial insult.

### Justice

In the adherence to this principle in this study, non-probability sampling was used. Purposive sampling was used to choose the nursing population. Confidentiality was assured by assigning numbers rather than names to questionnaires. Thus, it can be seen that the researcher applied the principle of justice in this study for all the stages. The researcher acknowledged all references. Proper citations have been given wherever warranted and a full bibliography was submitted in accordance with the policies and guidelines of the University of South Africa (UNISA) to avoid plagiarism.

### Reliability

According to Pera and Van Tonder (2017:382), reliability is a degree to which a measure of a concept is stable. To increase reliability, the questionnaire in this study was pre-tested on participants who did not participate in the main study. Moreover, precise and cautious wording of each question to avoid vagueness and leading respondents to particular answers ensured the reliability of the questionnaire. The respondents were also informed of the purpose of the study and the need to answer honestly. The questionnaire was evaluated and approved by the study supervisor and a statistician. The researcher compiled a preliminary questionnaire and then consulted an expert before compiling the final questionnaire.

### Validity

In this study, the researcher considered face and content validity (Gray et al. 2017:376). According to Gray et al. (2017:375) while examining validity, the types of evidence examined are based on response processes, internal structure, relationships on other variables or based on consequences of testing. Creswell and Creswell (2018:251) posited that validity refers to whether inferences can be made on a particular instrument. In this research, the researcher consulted the literature and obtained critique from experts when developing the questionnaire to achieve high quality data and to ensure that the questions meet the objectives.

## Results and discussion

The respondents were drawn from a personnel list obtained from the personnel department. A sample of 196 possible respondents, meeting the inclusion criteria, received a questionnaire (see Table 1).

**TABLE 1:** Number of respondents from the selected hospital.

Possible respondents	Response rate	Response percentage
196	162/196	82.7%

### Demographic data

**FIGURE 1:**
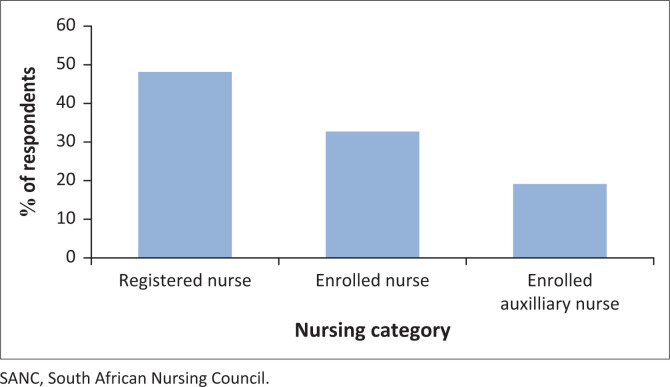
Respondents’ SANC registered titles (*n* = 162).

The study found that, of the respondents, 48.2% (*n* = 78) were registered nurses, whilst 32.7% (*n* = 53) were registered as staff nurse and 19.1% (*n* = 31) were registered as enrolled nursing auxiliaries (see Figure 1). According to the *Nursing Act*, Act No. 33 of 2005 (SANC 2005):

[*N*]o person may practice as a practitioner unless he or she is registered to practice in at least one of the following categories: Professional nurse, midwife, staff nurse, auxiliary nurse or auxiliary midwife. (p. 25)

**FIGURE 2:**
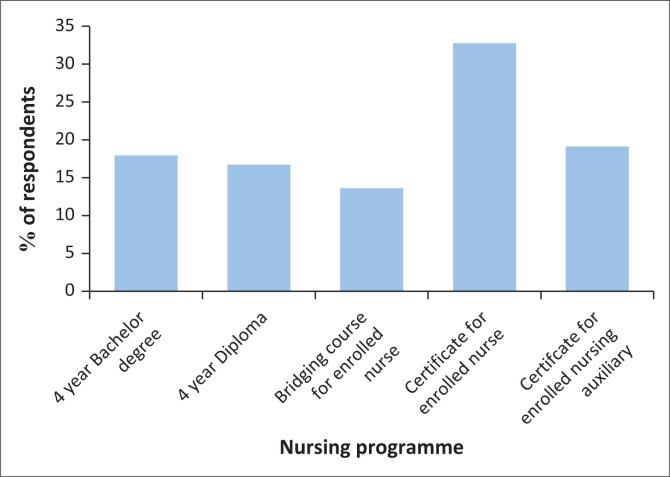
Type of programme followed (*n* = 162).

For the type of nursing undergraduate programme followed by the respondents, 17.9% (*n* = 29) had followed a 4-year bachelor degree, 16.7% (*n* = 27) had followed a 4-year diploma in general nursing, 13.6% (*n* = 22) had followed a 2-year bridging programme from enrolled nurse to general nurse diploma, 32.7% (*n* = 53) had followed a certificate for an enrolled nurse programme and 19.1% (*n* = 31) had followed a certificate for an enrolled nursing auxiliary programme (see Figure 2).

Blaauw, Ditlopo and Rispel (2014:5) found that the main nursing qualifications that have been recognised in South Africa since 1985 are Enrolled Nursing Auxiliary: 1-year certificate at a training hospital; Enrolled Nurse: 2-year certificate at nursing college; Registered Nurse: 4-year diploma at a nursing college or a 4-year baccalaureate degree at a university. Furthermore, the bridging programme also made it possible for enrolled nurses to finish a 2-year diploma at a nursing college and qualify as a registered nurse. This is consistent with this study, as this is the programmes that the respondents followed (see Figure 2).

### Role of the preceptor

**FIGURE 3:**
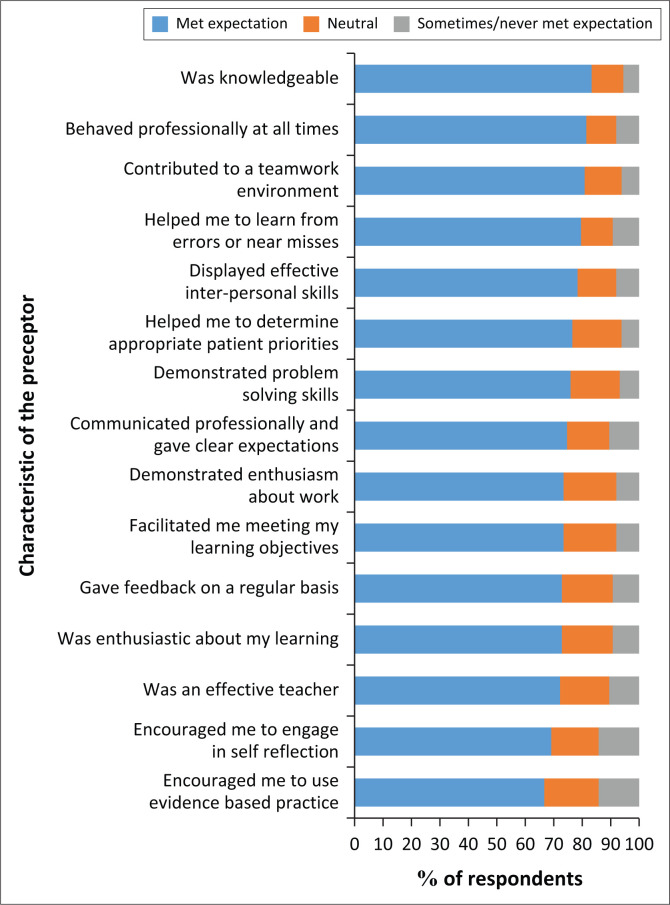
Roles of the preceptor (*n* = 162).

Respondents were asked about perceived roles and characteristics of the preceptor by indicating the following: always met expectation, frequently met expectation, neutral, sometimes met expectation or never met expectation (see Figure 3), in the following questions: The most significant aspects of the preceptor as indicated with the first data point in Figure 3 and had the highest percentage on, met expectation: Was knowledgeable 83.3%, behaved professionally 81.5%, contributed to a teamwork environment 80.9%, helped me to learn from errors or near misses: 79.6% indicated met expectation, displayed effective interpersonal skills: 78.4% indicated met expectation, helped me to determine appropriate patient priorities: 76.5% indicated met expectation and demonstrated problem solving skills 75.9%.

The lowest percentage with regard to the expectation of the preceptor role is displayed in the last two horizontal bars of Figure 3. In each of the individual bars it is indicated in the first data point. The expectations with the lowest percentage were: encouraged me to engage in self-reflection 69.1% and encouraged me to use evidence-based practice 66.7%. In this study, therefore, expectations were most frequently met at > 80% *n* = 129, and it was found that expectations were least frequently met at < 60%, *n* = 97.

In an analytical cross-sectional study conducted by Bahmanbijari et al. (2017:4129) to determine the viewpoint of students within the division of health, regarding the roles and features of a clinical role model at Kerman University of Medical Sciences in Iran, with 185 participants, the authors found that the results of their study emphasised the importance of the characteristics and roles of a clinical teacher as perceived by the participants in their study. A descriptive comparative study was conducted by Omer, Suliman and Moola (2015:4) to define the prospects of nurse preceptors’ roles and responsibilities as held by preceptors and their preceptees and the results indicated the following roles as important: protector, evaluator, educator and facilitator. A descriptive cross-sectional study conducted by Teferra and Mengistu (2017:87) to assess the knowledge and attitude towards clinical preceptorship found that in questions concerning the features for selection of preceptors, the response rate to a question asking what the minimal education of a preceptor should be, 50.9% of the respondents indicated a bachelor’s degree and in response to a question about years of clinical experience, two years (70.4%) was the minimum. The given results are consistent with the findings of this research. By determining the roles and characteristics of preceptors, expectations were most frequently met for: knowledgeability, professionalism and contribution to teamwork. However, this study also determined the use of evidence-based practice and self-reflection by preceptors, and it was found that these expectations were least frequently met.

The role of the preceptor, the participants highest level of response, met expectation, are displayed in Table 2. The table displays the category with the highest value to the different aspects of the preceptor’s role.

**TABLE 2:** The highest values to the preceptor role as per participant category (*n* = 162).

Preceptor characteristics and role	Participant category	Value	
		*n*	%
Was an effective teacher	Registered nurse	28	36
Demonstrated enthusiasm about work	Registered nurse	31	40
Was knowledgeable	Enrolled nurse	37	70
Helped determine patient priorities	Registered nurse	32	41
Demonstrated problem solving skills	Registered nurse	35	45
Encouraged evidence-based practice	Registered nurse	26	33
Helped me to learn from errors and near misses	Registered nurse	29	37
Behaved professionally	Registered nurse	42	54
Feedback on a regular basis	Registered nurse	33	42
Communicated professionally	Enrolled nurse	32	60
Contributed to teamwork environment	Enrolled nurse	31	58
Was enthusiastic about my learning	Registered nurse	30	39
Facilitated meeting learning objectives	Registered nurse	33	42
Effective interpersonal skills	Registered nurse	33	42
Encouraged self-reflection	Enrolled nurse	28	53

Regarding the most helpful support from the preceptor, recommendation of preceptorship and in-service training 66.7% (*n* = 108) of the respondents indicated clinical support; 95.7% (*n* = 155) of the respondents indicated recommendation of preceptorship; 82.1% (*n* = 133) of the respondents indicated that they attend in-service training, whilst 21.0% (*n* = 34) of the respondents indicated that they do not need preceptorship because of in-service training (see Table 3).

**TABLE 3:** Support from preceptor.

Question	Category	*N*	%
Most helpful support from preceptor	Clinical	108	66.7
	Academic	40	24.7
	Personal	7	4.3
	Emotional	7	4.3
Would recommend preceptorship to newly qualified nurse	-	155	95.7
Attending in-service training	-	133	82.1
Do not need preceptorship because of in-service training	-	34	21.0

Table 4 displays the values of each nursing category within this study, as respondent to each question.

**TABLE 4:** Value of each nursing category.

Question	Type of support	RN (*n* = 78)	EN (*n* = 53)	ENA (*n* = 31)
Most helpful support from preceptor	Clinical	57	33	18
	Academic	18	14	8
	Personal	1	4	2
	Emotional	2	2	3
Would recommend preceptorship to newly qualified nurse	Answered yes	76	49	30
Attending in-service training	Answered yes	63	46	24
Do not need preceptorship because of in-service training	Answered yes	18	10	6

RN, registered nurse; EN, enrolled nurse; ENA, enrolled nurse auxiliary.

### Limitations

As the research was conducted at a Level II regional hospital in the Western Cape. therefore, the findings cannot be generalised. The setting under study is a teaching hospital, which provides care in a larger area than a rural hospital, but less than a Level III hospital. In addition, the findings may differ from what may be obtained from a Level III hospital. The study also involved only 162 respondents whose backgrounds, education levels and areas of assignment differed. Despite the limitations, the study provides valuable information that could help in the promotion of preceptorship.

### Recommendations

Benner’s (1982) Novice to Expert model gives advice for job and information growth in nursing practice. It shows that continuous development is paramount in maintaining quality service to clients. The support from organisational structures for ongoing development programmes such as preceptorship within the clinical area is vital in achieving a skilled workforce. Preceptorship programmes need to be implemented and introduced from onboarding of newly qualified nurses. Preceptors need to be identified and encouraged by line managers to fulfil the preceptor role to achieve the objectives of their specific assignment areas. Nominated preceptors need formal training to fulfil their role as preceptor effectively.

## Conclusion

This research described the perceptions of newly qualified nurses on the guidance by preceptors towards becoming experts in practice. The questionnaire variables were summarised by frequency, percentage tabulation and were illustrated using bar charts. The data analysis was carried out using SAS Software, version 9.3 for Windows.

The results of the research show that respondents had more positive experiences than negative. Respondents appeared to regard their skill levels and task competence as high across all five skill levels of the Novice to Expert model (Benner 1982). The respondents indicated that the role and characteristics of the preceptor expectations were met for knowledgeability, professionalism and contribution to team work. Furthermore, the results indicated that respondents would recommend preceptorship. Whilst, preceptorship training is recommended to all registered professional nurses working with students and beginner nurses.

Moreover, respondents felt that their learning environment was conducive to learning and the preceptor role was highly perceived. The results of this research also revealed that the preceptors had an important role in minimising the bad experiences generally experienced with transition by newly qualified nurses.

The results of this research addressed the objectives of this research and gave answers to the research questions. The recommendations of this research are grounded on the results and the literature was used to validate the findings of the study.

According to Van Graan and Williams (2017:276) basic nursing education is insufficient to prepare novice nurses to assume and fulfil their professional role. The need for newly qualified nurses to be clinically and academically supervised and guided is crucial to ensure that they can provide safe and competent care and enable them to continuously grow in the profession from being novices to becoming experts. Preceptorship is essential in leading novices on their journey towards becoming experts in their specific field of practice. To gain a better understanding of the issues, the investigator used Benner’s Novice to Expert model (Benner 1982) as a theoretical framework to gain insight into the phenomenon under study.

Preceptorship is one of the major interventions available to support newly qualified nurses by easing the transition to practicing nurse and reducing the theory-practice gap. It is used for onboarding and to increase confidence, skill levels and expertise with the guidance of a preceptor.

## References

[CIT0001] Arbabi, H., Johnson, J. & Forgrave, D., 2018, ‘The effect of preceptorship on nurses training and preparation with implications for Qatar: A literature review’, *Journal of Nursing Education and Practice* 8(7), 44–50. 10.5430/jnep.v8n7p44

[CIT0002] Bahmanbijari, B., Beigzadeh, A., Etminan, A., Najarkolai, A.R., Khodaei, M. & Askari, S., 2017, ‘The perspective of medical students regarding the roles and characteristics of a clinical role model’, *Electronic Physician* 9(4), 4124–4130. 10.19082/4124 28607645 PMC5459282

[CIT0003] Bengtsson, M. & Carlson, E., 2015, ‘Knowledge and skills needed to improve as preceptor: Development of a continuous professional development course – A qualitative study Part 1’, *BioMed Central Nursing* 14, 51. 10.1186/s12912-015-0103-9 26478717 PMC4609157

[CIT0004] Benner, P., 1982, ‘From novice to expert’, *American Journal of Nursing* 82(3), 402–407. 10.2307/3462928 6917683

[CIT0005] Blaauw, D., Ditlopo, P. & Rispel, L.C., 2014, ‘Nursing education reform is South Africa: Lessons from a policy analysis study’, *Global Health Action* 7(1), 26401. 10.3402/gha.v7.26401 25537941 PMC4275647

[CIT0006] Blegen, M.A., Spector, N., Ulrich, B.T., Lynn, M.R., Barnsteiner, J. & Silvestre, J., 2015, ‘Preceptor support in hospital transition to practice programs’, *Journal of Nursing Administration* 45(12), 642–649. 10.1097/NNA.0000000000000278 26565643

[CIT0007] Bolt, J., Baranski, B., Bell, A. & Semchuk, W.M., 2016, ‘Assessment of preceptor development strategies across Canadian pharmacy residency programs’, *Canadian Journal of Hospital Pharmacy* 69(2), 144–148. 10.4212/cjhp.v69i2.1542 PMC4853182 27168636

[CIT0008] Botma, Y., 2016, ‘Suggested competencies for a preceptor training programme’, *Trends in Nursing* 3(1), 1–12. 10.14804/3-1-16

[CIT0009] Botma, Y., Jeggels, J. & Uys, L.R., 2012, ‘Preparation of clinical preceptors’, *Trends in Nursing* 1(1), 1–12. 10.14804/1-1-25

[CIT0010] Chapman, P.J., 2017, ‘The preceptorship experience of associate degree nursing students’, Unpublished Master’s dissertation, Indiana University of Pennsylvania, Indiana, Pa.

[CIT0011] Chong, H.C., Francis, K., Cooper, S. & Abdullah, K.L., 2014, ‘Current continuing professional education practice among Malaysian nurses’, *Nursing Research and Practice* 2014, 126748. 10.1155/2014/126748 24523961 PMC3913040

[CIT0012] Cloete, I.S. & Jeggels, J., 2014, ‘Exploring nurse preceptors’ perceptions of benefits and support of and commitment to the preceptor role in the Western Cape’, *Curationis* 37(1), 1–7. 10.4102/curationis.v37i1.1281 25685992

[CIT0013] Creswell, J.W. & Creswell, J.D., 2018, Research design: Qualitative, quantitative and mixed methods approaches, 5th edn., SAGE, London.

[CIT0014] Dahlke, S., O’Connor, M., Hannesson, T. & Cheetham, K., 2016, ‘Understanding clinical nursing education: An exploratory study’, *Nurse Education in Practice* 17, 145–152. 10.1016/j.nepr.2015.12.004 26775165

[CIT0015] Davis, A. & Maisano, P., 2016, ‘Patricia benner: Novice to expert – A concept whose time has come (again)’, *Oklahoma Nurse* 61(3), 13–15.

[CIT0016] Dhai, A., 2014, ‘The research ethics evolution: From Nuremberg to Helsinki’, *South African Medical Journal* 104(3), 178–180. 10.7196/SAMJ.7864 24897818

[CIT0017] Dias, J.M., Kurji, Z., Gulamani, S. & Mithani, Y., 2015, ‘Moving towards preceptorship model and blended learning methodologies in baccalaureate nursing education in Pakistan’, *International Journal of e-Healthcare Information Systems* 2(1), 51–55. 10.20533/ijehis.2046.3332.2015.0007

[CIT0018] Dornblaser, E.K., Ratka, A., Gleason, S.E., Ombengi, D.N., Tofade, T., Wigle, P.R. et al., 2016, ‘Current practices in global/international advanced pharmacy practice experiences: Preceptor and student considerations’, *American Journal of Pharmaceutical Education* 80(3), 1–11. 10.5688/ajpe80339 27170810 PMC4857634

[CIT0019] Dreyfus, S.E. & Dreyfus, H.L., 1980, *A five-stage model of the mental activities involved in directed skill acquisition*, University of California, Berkley, CA.

[CIT0020] Foote, E.F., Roland, B.E., Gionfriddo, M.R. & Holt-Macey, S., 2014, ‘Differences between residency- and non-residency-trained preceptors on student perceptions and activities of community practice advanced pharmacy practice experiences’, *Current in Pharmacy Teaching and Learning* 6(2), 259–264. 10.1016/j.cptl.2013.11.006

[CIT0021] Gray, J.R., Grove, S.K. & Sutherland, S., 2017, *Burns and Grove’s: The practice of nursing research: Appraisal, synthesis and generation of evidence*, 8th edn., Elsevier, Maryland Heights, MO.

[CIT0022] Hall, K.C., 2016, ‘Role functions of staff nurse preceptors for undergraduate pre-licensure nursing students’, *Journal of Nursing Education and Practice* 6(7), 19–30. 10.5430/jnep.v6n7p19

[CIT0023] Hickerson, K.A., Terhaar, M.F. & Taylor, L.A., 2016, ‘Preceptor support to improve nurse competency and satisfaction: A pilot study of novice nurses and preceptors in a pediatric intensive care unit’, *Journal of Nursing Education and Practice* 6(12), 57–62. 10.5430/jnep.v6n12p57

[CIT0024] Hilaire, D.M. & Sheldon, L.K., 2015, ‘Development of communication skills in healthcare: Perspectives of new graduates of undergraduate nursing education’, *Journal of Nursing Education and Practice* 5(7), 30–37. 10.5430/jnep.v5n7p30

[CIT0025] Hofler, L. & Thomas, K., 2016, ‘Transition of new graduate nurses to the workforce: Challenges and solutions in the changing health care environment’, *North Carolina Medical Journal* 77(2), 133–136. 10.18043/ncm.77.2.133 26961840

[CIT0026] Jeggels, J., Traut, A. & Africa, F., 2013, ‘A report on the development and implementation of a preceptorship training programme for registered nurses’, *Curationis* 36(1), 1–6. 10.4102/curationis.v36i1.106 26697619

[CIT0027] Jones, S., 2015, ‘Understanding the experience and perceptions of managers and preceptors involved in competency assessment and performance management of nursing staff identified as practicing unsafely: An evaluation of the effectiveness of the SIP/PIP framework’, Unpublished Master’s dissertation, University of Otago, Otago, NZ.

[CIT0028] Koetting, C., Ecuyer, K.L. & Benz, M., 2015, ‘Nurse practitioner student preceptor orientation via a wiki’, *Journal of Nursing Education and Practice* 5(2), 124–130. 10.5430/jnep.v5n2p124

[CIT0029] Lalonde, M. & Hall, L.M., 2017, ‘Preceptor characteristics and the socialization outcomes of new graduate nurses during a preceptorship programme’, *Nursing Open* 4, 24–31. 10.1002/nop2.58 28078096 PMC5221437

[CIT0030] Lennox, S., Skinner, J. & Foureur, M., 2008, ‘Mentorship, preceptorship and clinical supervision: Three key processes for supporting midwives’, *New Zealand College of Midwives Journal* 39, 7–12.

[CIT0031] Lewis, S. & McGowan, B., 2015, ‘Newly qualified nurses’ experience of a preceptorship’, *British Journal of Nursing* 24(1), 40–43. 10.12968/bjon.2015.24.1.40 25541875

[CIT0032] Makie, V., 2021, *email*, 03 February, warriodenehansen@gmail.com.

[CIT0033] Miller, J., Vivona, B. & Roth, G., 2016, ‘Nursing preceptors and meaning making’, *Qualitative Report* 21(11), 2014–2032, viewed 21 February 2018, from https://nsuworks.nova.edu/tqr/vol21/iss11/7.

[CIT0034] Missen, K., McKenna, L. & Beauchamp, A.J., 2014, ‘Satisfaction of newly graduated nurses enrolled in transition-to-practice programs in their first year of employment: A systematic review’, *Journal of Advanced Nursing* 70(11), 2419–2433. 10.1111/jan.12464 24989716

[CIT0035] Morgan, M., 2017, ‘Improving new nurses’ transition to practice’, Unpublished doctoral thesis, Walden University, Minneapolis, MN.

[CIT0036] Omer, T.A., Suliman, W.A. & Moola, S., 2015, ‘Roles and responsibilities of nurse preceptors: Perceptions of preceptors and preceptees’, *Nurse Education in Practice* 1(1), 1–6. 10.1016/j.nepr.2015.07.005 26255079

[CIT0037] Owens, N.G., 2013, ‘New graduate nurse preceptor program: A collaborative approach with academia’, *Journal of Nursing Education and Practice* 3(12), 1–9. 10.5430/jnep.v3n12p1

[CIT0038] Padayachee, P., 2014, ‘The role of the clinical preceptor in enhancing nursing education at a nursing college’, Unpublished Master’s dissertation, Stellenbosch University, Stellenbosch.

[CIT0039] Peixoto, L.S., Tavares, C.M. & De Queiroz, P.P., 2014, ‘Research about the knowledge and teaching practice of the preceptor: A test pilot’, *Journal of Nursing* 8(7), 2038–2046.

[CIT0040] Pera, S.A. & Van Tonder, S., 2017, *Ethics in healthcare*, 3rd edn., Juta, Landsdowne.

[CIT0041] Polit, D.F. & Beck, C.T., 2017, *Nursing research: Generating and assessing evidence for nursing practice*, 10th edn., Wolters Kluwer, Philadelphia, PA.

[CIT0042] Quek, G. & Shorey, S., 2018, ‘Perceptions, experiences, and needs of nursing preceptors and their preceptees on preceptorship: An intergrative review’, *Journal of Professional Nursing* 34(1), 417–428. 10.1016/j.profnurs.2018.05.003 30243699

[CIT0043] Rikhotso, S.R., Williams, M.J.S. & De Wet, G., 2014, ‘Student nurses’ perceptions of guidance and support in rural hospitals’, *Curationis* 37(1), 1–6. 10.4102/curationis.v37i1.1164 26852424

[CIT0044] Rogan, E., 2014, ‘Preparation of nurses who precept baccalaureate nursing students: A descriptive study’, *Journal of Continuing Education in Nursing* 40(12), 565–570. 10.3928/00220124-20091119-06 20000266

[CIT0045] Ryan, C. & McAllister, M., 2016, ‘Enrolled nurses’ experiences learning the nurse preceptor role: A qualitative evaluation’, *Journal of Australian College of Nursing* 24(3), 267–273. 10.1016/j.colegn.2016.04.001

[CIT0046] Silvestre, J., Ulrich, B.T., Johnson, T., Spector, N. & Blegen, M.A., 2017, ‘A multisite study on a new graduate registered nurse transition to practice program: Return on investment’, *Nursing Economics* 35(3), 110–118.

[CIT0047] Sodeify, R., Vanaki, Z. & Mohammadi, E., 2013, ‘Nurses’ experiences of perceived support and their contributing factors: A qualitative content analysis’, *Iran Journal of Nursing and Midwifery Research* 18(3), 191–197. PMC3748536 23983753

[CIT0048] South African Nursing Council (SANC), 2005, *Nursing Act No. 33*, South African Nursing Council, Juta, South Africa.

[CIT0049] Teferra, A.A. & Mengistu, D., 2017, ‘Knowledge and attitude towards nursing clinical preceptorship among Ethiopian nurse educators: An institution-based cross-sectional study’, *International Journal of Africa Nursing Sciences* 7, 82–88. 10.1016/j.ijans.2017.10.001

[CIT0050] Van Graan, A.C. & Williams, M.J.S., 2017, ‘A conceptual framework to facilitate clinical judgement in nursing: A methodological perspective’, *Health South Africa* 22(11), 275–290. 10.1016/j.hsag.2017.01.004

[CIT0051] Webster, A.L., 2016, ‘Preceptor program for new graduate nurses’, Unpublished doctoral study, Walden University, Minneapolis, MN.

[CIT0052] Whitehead, B., Owen, P., Holmes, D., Beddingham, E., Simmons, M., Henshaw, L. et al., 2013, ‘Supporting newly qualified nurses in the United Kingdom: A systematic literature review’, *Nurse Education Today* 33(4), 370–377. 10.1016/j.nedt.2013.01.009 23416083

[CIT0053] Zaayman, L.S., 2016, ‘Professional nurses’ experiences of their community service placement year at a secondary academic hospital in the Western Cape’, Unpublished Master’s dissertation, University of the Western Cape, Cape Town.

[CIT0054] Zerwekh, J. & Garneau, A.Z., 2018, *Nursing today: Transition and trends*, 9th edn., Elsevier, Maryland Heights, MO.

